# Sit-to-Stand Performance, Moderate-to-Vigorous Physical Activity, and Health-Related Quality of Life in Knee Osteoarthritis: A Prospective Mediation Analysis

**DOI:** 10.5888/pcd23.250405

**Published:** 2026-04-23

**Authors:** Kristen Dean, Donya Nemati, Ruopeng Sun, Matthew Smuck, Junichi Kushioka, Thomas M. Best, Navin Kaushal

**Affiliations:** 1 University of Miami, Miller School of Medicine, Miami, Florida; 2 College of Nursing, The Ohio State University, Columbus, Ohio; 3 Department of Orthopaedic Surgery, Stanford University, Stanford, California; 4 Spine and Scoliosis Center, Shonan Fujisawa Tokushukai Hospital, Kanagawa, Japan; 5 Department of Orthopaedic Surgery, Osaka University, Osaka, Japan; 6 Department of Orthopedics, University of Miami, Miami, Florida; 7 UHealth Sports Medicine Institute, University of Miami, Miami, Florida; 8 Department of Health Sciences, School of Health and Human Sciences, Indiana University, Indianapolis, Indiana

## Abstract

**Introduction:**

Knee osteoarthritis (KOA) is the most common form of arthritis, leading to illness, decreased physical function, and reduced health-related quality of life (HR-QoL). Sit-to-stand (STS) testing is a commonly used measure of physical capacity in KOA. This study examined whether STS performance and moderate-to-vigorous physical activity (MVPA) helped account for the association between KOA pain and subsequent HR-QoL.

**Methods:**

Data across 2 years (at year 6 and year 8 visits) were used from the Osteoarthritis Initiative (OAI), a multisite, longitudinal study of people with or at risk for KOA. We used serial mediation analysis to examine whether year 6 STS performance and year 8 MVPA accounted for the association between year 6 KOA pain and year 8 HR-QoL, adjusting for age, sex, and race.

**Results:**

Significant correlations among all model variables supported model testing. The serial mediation model revealed that an increase in KOA pain predicted worse STS performance (β = .15; *P* < .001). MVPA was reduced by an increase in KOA pain (β = −.13; *P* < .001) but not STS (β = −.02; *P* = .46). MVPA subsequently predicted HR-QoL (β = .12; *P* < .001). The overall indirect pathway was significant (β = −0.02; 95% CI, −0.040 to −0.009), but the direct pathway also remained significant (β = −0.47; 95% CI, −0.536 to −0.410), denoting a partial mediation effect.

**Conclusion:**

STS performance and MVPA partially mediated the relationship between KOA pain and HR-QoL, highlighting mechanisms to address in future interventions for improving HR-QoL. These results support STS as a complementary tool to pain for understanding MVPA participation and HR-QoL in KOA patients. The findings also underscore the importance of integrating self-management strategies to address pain in behavior-change interventions.

**Summary**

**What is already known about this topic?**

Knee osteoarthritis (KOA) pain and physical activity are both associated with health-related quality of life (HR-QoL). Sit-to-stand (STS) testing is a clinical measure of lower limb strength commonly used in people with KOA.

**What is added by this report?**

STS performance and physical activity partially mediated the relationship between KOA pain and HR-QoL, highlighting mechanisms to test in future interventions aimed at improving HR-QoL for patients with KOA. These findings support the utility of STS testing as a complementary tool to pain assessment to better understand participation in moderate-to-vigorous physical activity and compromised HR-QoL in patients with KOA.

**What are the implications for public health practice?**

The findings explain some mechanisms of why pain compromises HR-QoL among people with KOA. Behavioral strategies to alleviate and cope with pain could help promote exercise to improve HR-QoL in patients with KOA.

## Introduction

Knee osteoarthritis (KOA) is the most prevalent form of arthritis worldwide, characterized by high disability rates and illness (1). A common symptom of KOA progression is pain, which can limit physical performance and may compromise balance. The sit-to-stand (STS) test primarily evaluates lower body strength, balance, and physical capacity by measuring the time required to repeatedly rise from and return to a seated position. STS testing has been used in people with KOA and is attractive for clinical settings because it is brief, feasible, and reproducible (2). STS tests have been used in large-scale cohort studies, such as the Osteoarthritis Initiative (OAI), and in clinical trials (2,3). In addition to fitness assessments, STS performance is a predictor of physical activity, where exercise is a cornerstone for managing KOA (4–6).

Regularly engaging in physical activity, specifically moderate-to-vigorous physical activity (MVPA), is one of the most effective countermeasures for KOA symptom control. The data support the use of MVPA to reduce pain and enhance physical functioning, supporting the ability to perform activities of daily living and thereby promoting better health-related quality of life (HR-QoL) (5,6). HR-QoL is a multidimensional construct that reflects a person’s overall well-being as it relates to health, encompassing physical, psychological, and social functioning. Specifically, HR-QoL captures the effect of health status, including symptoms and disability, on daily activities, participation, and perceived life satisfaction. Hence, maintaining or improving HR-QoL is often recognized as the goal among patients with chronic conditions, such as KOA.

Collectively, prior research supports relationships between KOA pain with HR-QoL and STS performance, STS performance with MVPA, and MVPA with HR-QoL. The supported arrangement of these variables and relationships is depicted in a proposed serial-mediation model (Figure). The model situates STS performance and MVPA as mediators of the relationship between KOA pain and HR-QoL. The mediation model allows us to test the pathway through which KOA pain affects HR-QoL, with STS performance and MVPA acting as sequential mediators. The STS test can be performed quickly in a clinical setting so physicians can provide immediate assessments to inform patients, and it can be a helpful measurement in rural clinics and low-income neighborhoods. The relationship between STS assessment and physical activity is also important because it can provide insight to physicians on why their patients may not be physically active, a factor that is predictive of HR-QoL.

**Figure:**
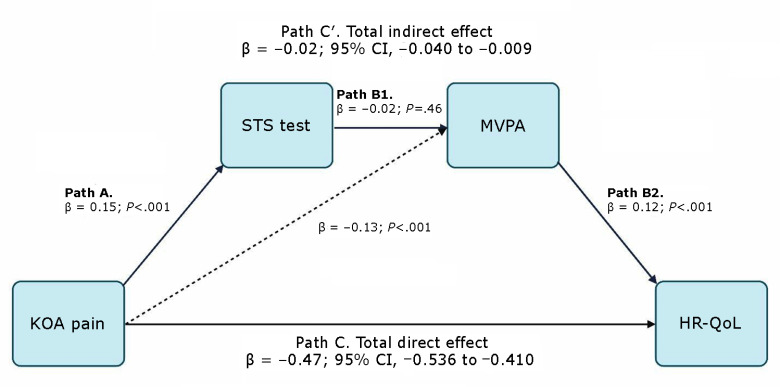
Proposed serial mediation model: direct and indirect pathways of KOA pain and HR-QoL. Abbreviations: HR-QoL, health-related quality of life; KOA, knee osteoarthritis; MVPA, moderate-to-vigorous physical activity; STS, sit-to-stand.

The aim of this study was to examine whether STS performance and MVPA account for the association between KOA pain and subsequent HR-QoL by using prospective OAI data collected across 2 years. The primary purpose was to test the correlations among proposed model variables. The supported relationships would allow testing of the secondary objective, which is the proposed serial mediation model. We hypothesized that KOA pain would predict STS performance (path A), that STS would predict MVPA (path B1), and that MVPA would predict HR-QoL (path B2). Finally, we hypothesized that these pathways would mediate the relationship to explain why people with greater KOA symptoms experience lower HR-QoL (path Cʹ) (Figure).

## Methods

### Participants

The OAI is a multicenter, longitudinal prospective and observational study of US patients who have KOA or who are determined to be at risk of developing KOA. The study is a public–private partnership between the National Institutes of Health and private industry that was established to develop a public-domain research resource to study KOA onset, progression, and biomarkers through biospecimen collection associated with this condition. Participants aged 45 to 79 years were enrolled from February 2004 to May 2006 and followed longitudinally (3,7). Retention remained high, with approximately 7% of participants lost after 5 years and no-contact rates stabilizing at 15% to 18% (3). The cohort completed repeated in-clinic visits with the OAI research team, including baseline through 48 months, and additional 72-month and 96-month imaging visits. Mailed or telephone assessments were conducted at 60 and 84 months (3). Accordingly, the OAI database was chosen for the current study for its comprehensive and longitudinal design, with capabilities of offering insights into complex interaction of variables associated with the patient experience of KOA.

We conducted a secondary analysis of OAI data from the year 6 and year 8 visits, the only 2 visits that included accelerometer data. People with a complete dataset at both the baseline and the 2-year follow-up visit (valid clinical, functional, accelerometry, and radiographic data) were included. People who had knee surgery between assessments were then excluded. After applying these criteria, 782 participants remained for analysis (226 progression cohort, 551 incident cohort, and 5 control cohort at OAI enrollment). The original OAI study obtained institutional review board approval and informed consent. The current study used de-identified, publicly available data and therefore was exempt from additional review.

### Measures

KOA pain was operationalized by using the Western Ontario and McMaster Universities Osteoarthritis Index (WOMAC) score over 2 years. The WOMAC is a questionnaire in which participants respond to items related to their level of perceived difficulty across 24 metrics, across 3 domains: pain (5 items), stiffness (2 items), and physical function (17 items). Each item was rated using a Likert scale, with corresponding numbers 0 through 4. The total score range for each category was 0 to 20 for pain, 0 to 8 for stiffness, and 0 to 68 for physical function. The WOMAC was chosen as a measure of KOA pain for its validated and standardized use in both research and clinical settings for KOA patients (8).

STS testing measures the time required for a person to stand up and sit down 5 consecutive times (2,9). When performing this test, the participant is instructed to begin seated in a chair (43–46 cm in height), with both arms crossed over the chest and back flush against the back of the chair (2,10). To start STS testing, the participant rises from the seated position, standing up completely before returning to the seated position. The participant is asked to perform this action 5 consecutive times as quickly as possible. STS performance has been noted as a useful, low-cost, and consistent tool for measuring lower limb muscle strength, providing rationale for the use of STS testing as a viable metric in this study (2,10,11).

MVPA was measured by using the ActiGraph GT1M monitor (ActiGraph LLC), a hip-mounted accelerometer validated for accuracy in adults, including those with KOA. Participants wore the accelerometer for 7 consecutive days at each visit to estimate weekly minutes of MVPA. Data accuracy was ensured by identifying nonwear periods as intervals of 90 minutes or more without activity (12). A minimum wear time of 10 hours per day for 4 to 7 days was required for inclusion (13).

HR-QoL assesses individual health capacity, and The Short Form Health Survey 12 (SF-12) was administered to estimate HR-QoL (14). The SF-12 assessed several domains of health, such as general health, physical functioning, mental health, and activity limitations that stem from either mental or physical deficits. Participants were asked to consider their experience over the past month when answering the survey. Responses were scored on a 5-point Likert scale (not at all [1], a little bit [2], moderately [3], quite a bit [4], extremely [5]). Reverse scoring was used for 4 of the items on the SF-12 (14). Following scoring guidelines, raw scores were converted to T-scores 50 + ([(score − mean) ∕ SD] × 10) to yield a score range of 0 to 100, where higher scores correspond with better HR-QoL (15–18).

### Analysis

Bivariate correlations were used to assess significant differences among these variable associations at baseline and follow-up (Table 1). Significant differences in outcome variables were further tested in serial mediation analyses to determine the role of STS testing. IBM SPSS Statistics version 31 (International Business Machines Corp) was used to perform these statistical analyses.

**Table 1:** Zero-Order Bivariate and Model Correlations Among Study Variables, Osteoarthritis Initiative Study

Variable	Pain	STS	MVPA	HR-QoL	Age	Sex	Race
1. Pain	1	.128^c^	−.153^d^	−.508^d^	−.021	.059	−.212^c^
2. STS		1	.087^e^	−.111^c^	.112^c^	.040	−.041
3. MVPA			1	.092	−.421^c^	−.186^c^	−.094^c^
4. HR-QoL				1	−.049	.089^e^	.138^c^
5. Age					1	.093^e^	−.093^e^
6. Sex^a^						1	.120^c^
7. Race^b^							1

Abbreviations: HR-QoL, health-related quality of life; MVPA, moderate-to-vigorous physical activity; STS, sit-to-stand.

a Sex was self-reported and coded as 1 = male and 2 = female.

b Race was self-reported and coded as 1 = White/Caucasian, 2 = Black/African American, 3 = Asian, and 4 = Other (including mixed race or not specified).

c *P* < .001.

d *P* < .001.

e *P* < .05.

Serial mediation analysis was conducted using the PROCESS macro (version 4.1) in SPSS, testing whether STS performance and MVPA could explain the relationship between KOA pain and patient HR-QoL (19). Mediation analysis tests whether the effect of an independent variable (X) on a dependent variable (Y) operates, or is explained through, 1 or more intermediate variables called mediators (M_1_, M_2_). For this study, the model tested if baseline STS score (M_1_) and time 2 MVPA (M_2_) mediated the relationship between baseline KOA pain (X) and time 2 HR-QoL (Y) (20). The Figure depicts the tested variables, portraying the direct and indirect pathways. Together, paths A, B1, and B2 make up the total indirect pathway (path Cʹ), whereas the direct pathway is the relationship between KOA pain and HR-QoL (path C). The model tested whether knee pain (KOA pain) predicted STS scores (path A), followed by whether STS scores predicted physical activity (MVPA; path B1), and whether physical activity predicted HR-QoL (path B2). Finally, the direct pathway tested whether knee pain predicted HR-QoL (path C). Successful mediation was denoted by a significant indirect pathway (path Cʹ) with a confidence interval not including zero. If the direct pathway (path C) remained significant, then this would indicate partial mediation. The model controlled for age, sex, and race.

## Results

### Participants

Among the population assessed in this study (n = 782), the average age was 64.95 years (SD, 9.07), 55.5% were female (n = 434), and 44.5% were male (n = 348), with an average body mass index of 28.54 (calculated as weight in kilograms divided by height in meters squared) (SD, 4.75) (Table 2). Most participants were of White or Caucasian descent (85.8%, n = 671), with Black or African American as the second largest proportion (13.1%, n = 102). The remaining population identified as Asian (0.5%, n = 4) or Other non-White (0.6%, n = 5). The mean pain score was 11.91 (SD, 14.90), mean STS score was 10.66 (SD, 4.14) seconds, mean MVPA was 19.68 (SD, 19.45) minutes per week, and mean HR-QoL rating was 103.5 (SD, 11.23). 

**Table 2:** Participant Characteristics and Descriptive Statistics, Osteoarthritis Initiative Study (n = 782)

Characteristic	Value (%)
**Age, y (SD)**	64.95 (9.07)
**Sex**	
Women, no. (%)	434 (55.5)
Men, no. (%)	348 (44.5)
**Body mass index, mean (SD)**	28.54 (4.75)
**Race, no. (%)**	
White or Caucasian	671 (85.8)
Black	102 (13.1)
Other^c^	9 (1.3)
**Tested variables**	
Pain score^a^, mean (SD)	11.91 (14.90)
Sit-to-stand score, mean (SD) seconds	10.66 (4.14)
Moderate-to-vigorous physical activity, mean (SD) minutes per week	19.68 (19.45)
Health-related quality of life rating^b^, mean (SD)	103.50 (11.23)

a Measured by using the Western Ontario and McMaster Universities Osteoarthritis Index (WOMAC) score (8).

b Measured by using the Short Form Health Survey 12 (14).

c 0.5% of the Other non-White accounts for Asian people.

### Conceptual model

Bivariate correlations showed significant associations among model variables. Baseline pain correlated with STS (*r* = .128, *P* < .001), MVPA (*r* = −.153, *P* < .001), and HR-QoL (*r* = −.508, *P* < .001) (Table 1)

The serial mediation model (Figure) found that worsening in KOA pain predicted slower (worsening) STS score (β = .15; *P* < .001). MVPA was reduced by increase in KOA pain (β = −.13; *P* < .001) but not STS (β = −.02; *P* = .46). MVPA subsequently predicted HR-QoL (β = .12; *P* < .001). The overall indirect pathway was significant (β = −0.02; 95% CI, −0.040 to −0.009), but the direct pathway also remained significant (β = −0.47; 95% CI −0.536 to −0.410), indicating a partial mediation effect.

## Discussion

This study examined whether STS performance and MVPA account for the association between KOA pain and subsequent HR-QoL. Overall, our findings indicated that STS and MVPA partially mediated the relationship between KOA pain and compromised HR-QoL, with STS emerging as a potential complementary measure of pain assessment. The tested model is supported by prior research, and to our knowledge, this is the first study to examine these relationships by using a serial mediation approach. Specifically, we investigated how KOA pain may influence HR-QoL through a sequential pathway involving STS performance and MVPA. This framework provides insight into the mechanisms linking symptoms to outcomes and highlights potential targets for intervention. Similar serial mediation approaches have been applied in other chronic disease contexts. For example, in type 2 diabetes, researchers have shown that health literacy influences glycemic control through empowerment and self-care behaviors, whereas in rheumatoid arthritis, continuity of care affects health outcomes via treatment adherence and disease activity (21,22). 

Bivariate correlations across both time points aligned with previous literature findings and supported the proposed arrangement of the serial mediation model (5,17,18,23). A notable finding was that KOA pain predicted all variables in the controlled model, functioning as a salient determinant compromising functionality, physical activity, and overall HR-QoL. Substantive data support the impact of pain as a deterrent for activities of daily living, which establishes its direct relationship to HR-QoL. However, despite STS performance being proximal to MVPA and demonstrating significant correlation, pain was noted to be a stronger predictor of MVPA behavior in a model that did not include STS performance as a mediator. These findings highlight the importance of model-based testing to identify magnitude effects of predictors in the context of relevant variables (24,25). On the translational front, these findings emphasize the importance of pain management as potentially a first line of self-management, which would increase STS score, promote MVPA, and subsequently improve HR-QoL (26).

People with increased KOA pain tended to perform worse on STS testing, confirming our hypothesis and underscoring that self-reported functional limitations align with objective biomechanical capacity. These findings are supported by Anan et al, who showed that women with KOA performed the STS test with significantly less mechanical efficiency compared with asymptomatic matched controls that did not meet KOA clinical criteria, largely because of pain and compensatory strategies (23). More recently, Jørgensen et al found that STS power predicted functional outcomes in patients with advanced OA, reinforcing its utility as a clinical metric in disease monitoring (27). In parallel, Yokota et al demonstrated that STS performance had a direct effect on HR-QoL among older adults with KOA, whereas Master et al confirmed its association with MVPA and functional independence (28,29). Collectively, these studies emphasize STS testing as a low-cost, reproducible, and complementary measure for KOA pain. STS testing provides complementary, objective biomechanical information to be used in conjunction with pain assessment. Specifically, STS offers 1) an objective, performance-based reflection of functional capacity beyond subjective report and 2) insight into the biomechanical consequences of pain, lower-limb strength, balance, and neuromuscular coordination that predict disability and fall risk independently of pain severity (27,30).

### Strengths and limitations

Our study has both strengths and limitations. The use of serial mediation modeling enabled simultaneous evaluation relationships of variables, reducing the risk of type II error while reflecting the dynamic interplay among KOA, physical function, activity behavior, and quality of life. The large sample size from a prospective study across 2 years was a strength, as were the use of established and validated measures, in addition to clinically measured STS performance and objectively measured MVPA. Limitations of this study were separate assessments of aerobic and strength training physical activity, as the latter yields better functionality among people with KOA (31). These 2 measures of exercise could also provide opportunities for identifying optimal amounts of each type of exercise and dose–response analysis. In addition, subsequent investigations should assess STS testing prospectively as a screening tool in KOA. These limitations also serve as factors to consider in future investigations.

### Conclusion

The serial mediation model contributes to understanding the mechanisms by which pain compromises HR-QoL among people with KOA. STS performance and exercise partially mediated the relationship between KOA pain and HR-QoL, highlighting mechanisms to improve in future interventions for improving HR-QoL. These findings support the use of STS testing as a complementary tool to pain assessment to understand participation in MVPA and compromised HR-QoL in patients with KOA. Our findings support future clinical trials to investigate whether promoting self-management behaviors for addressing pain, along with promoting MVPA, would contribute toward improving HR-QoL among patients with KOA.
